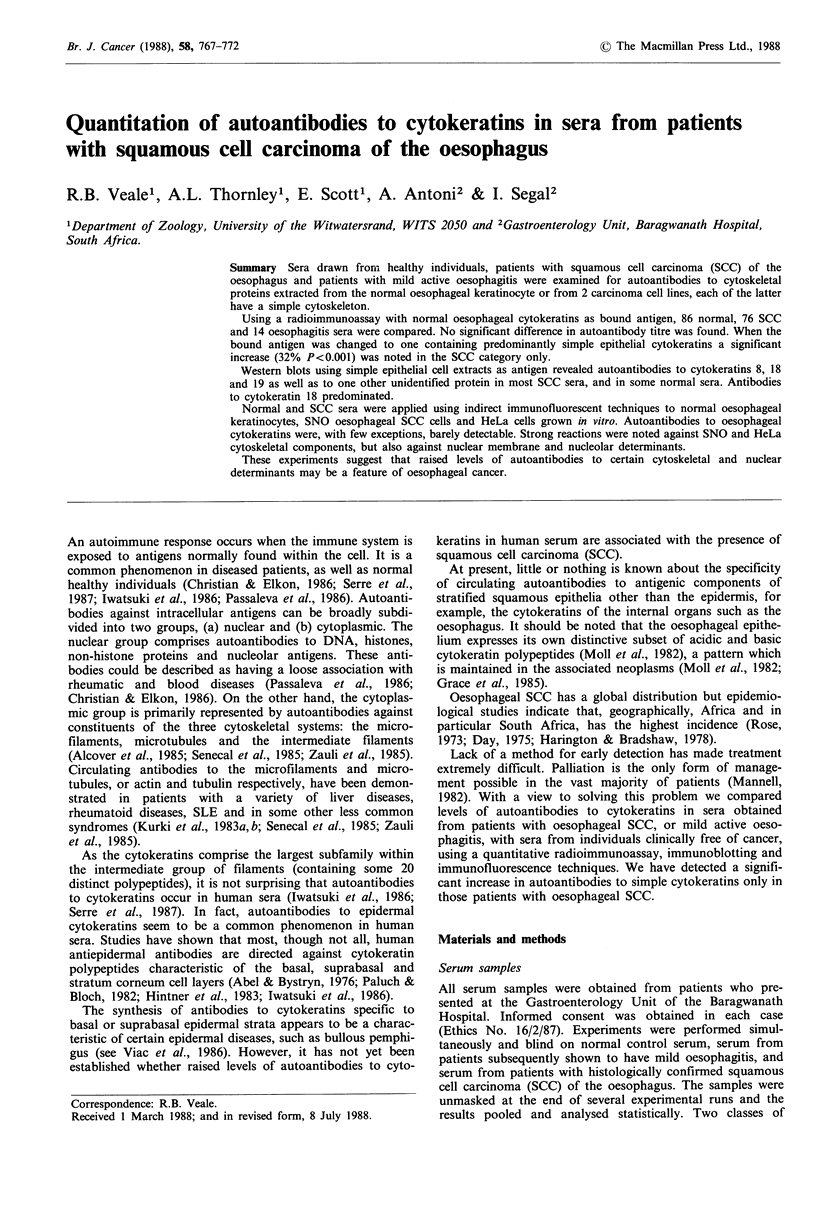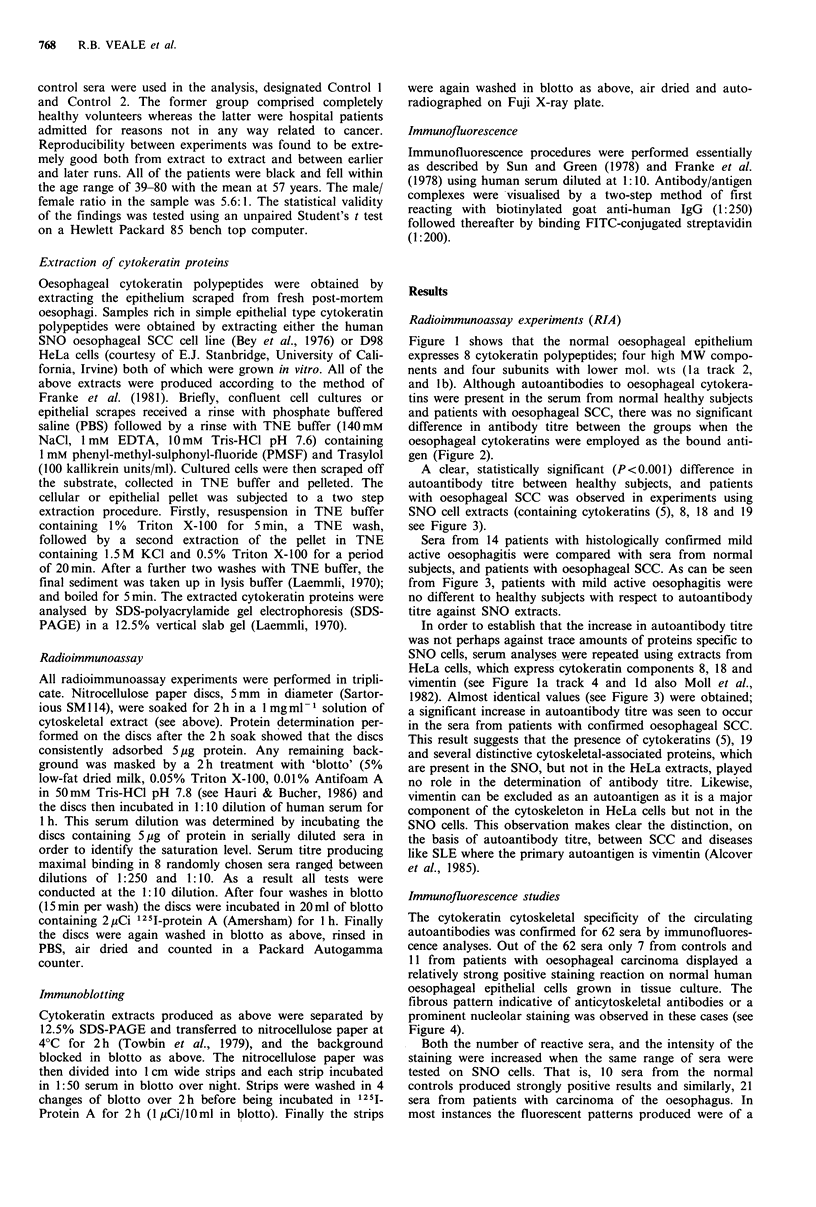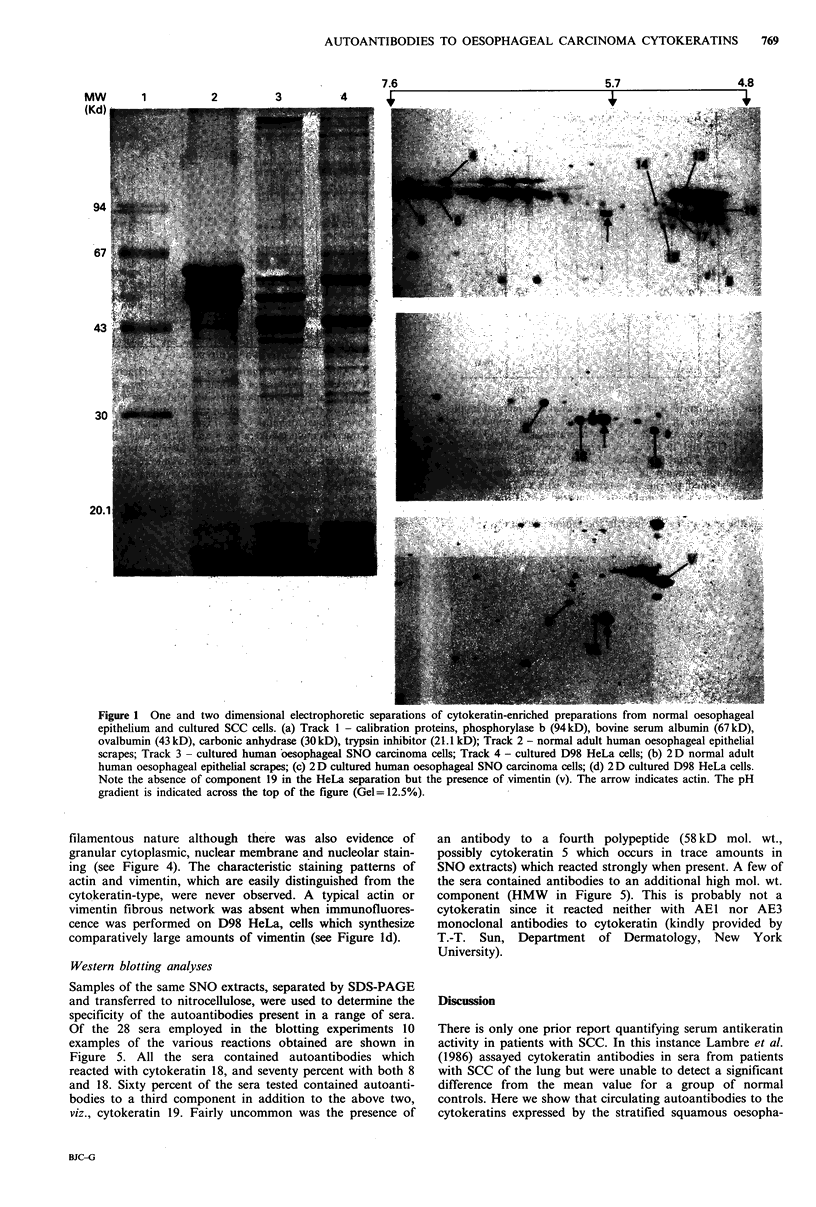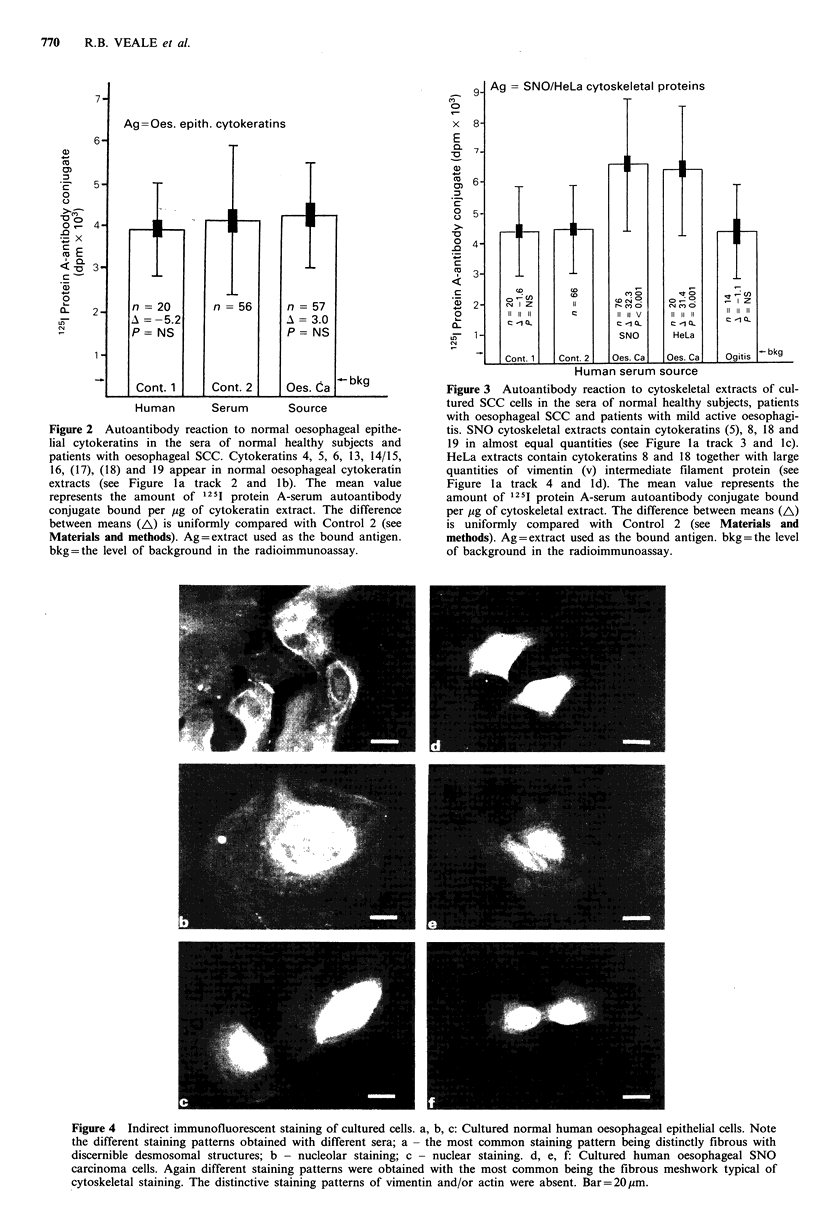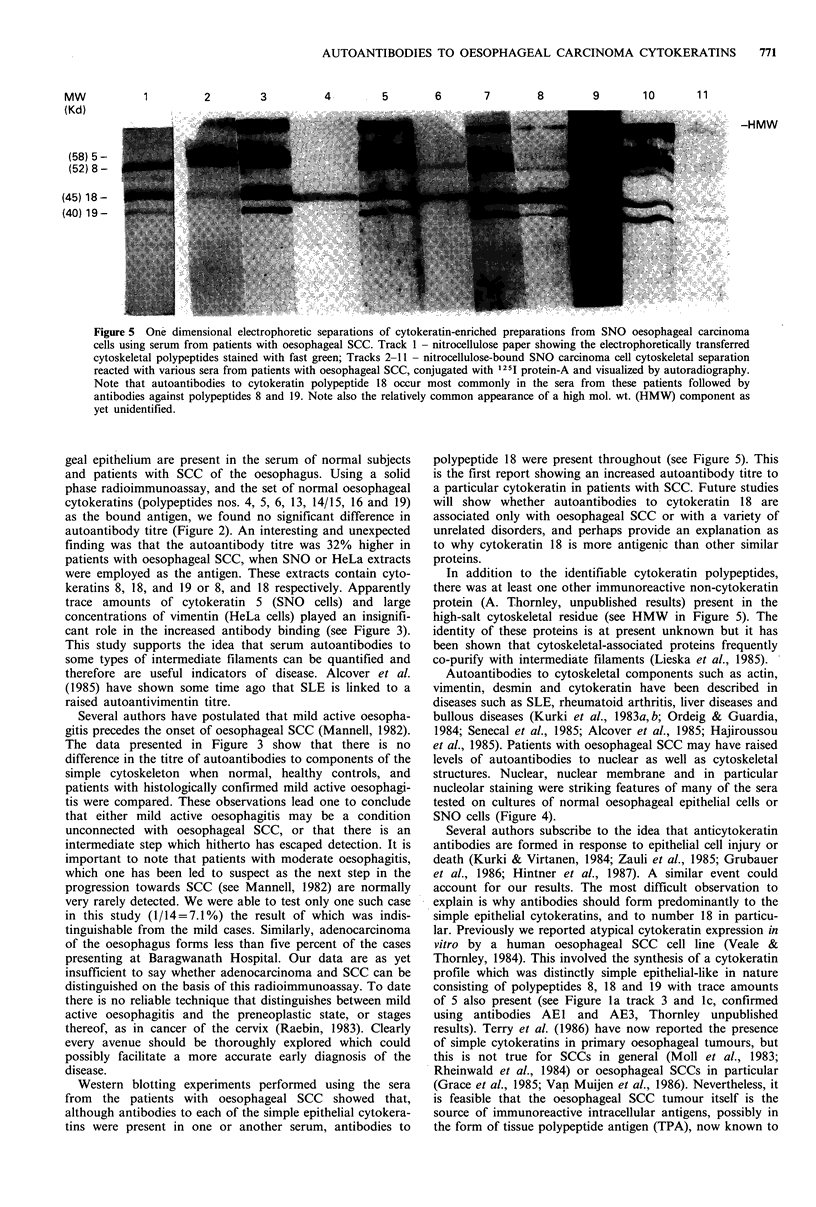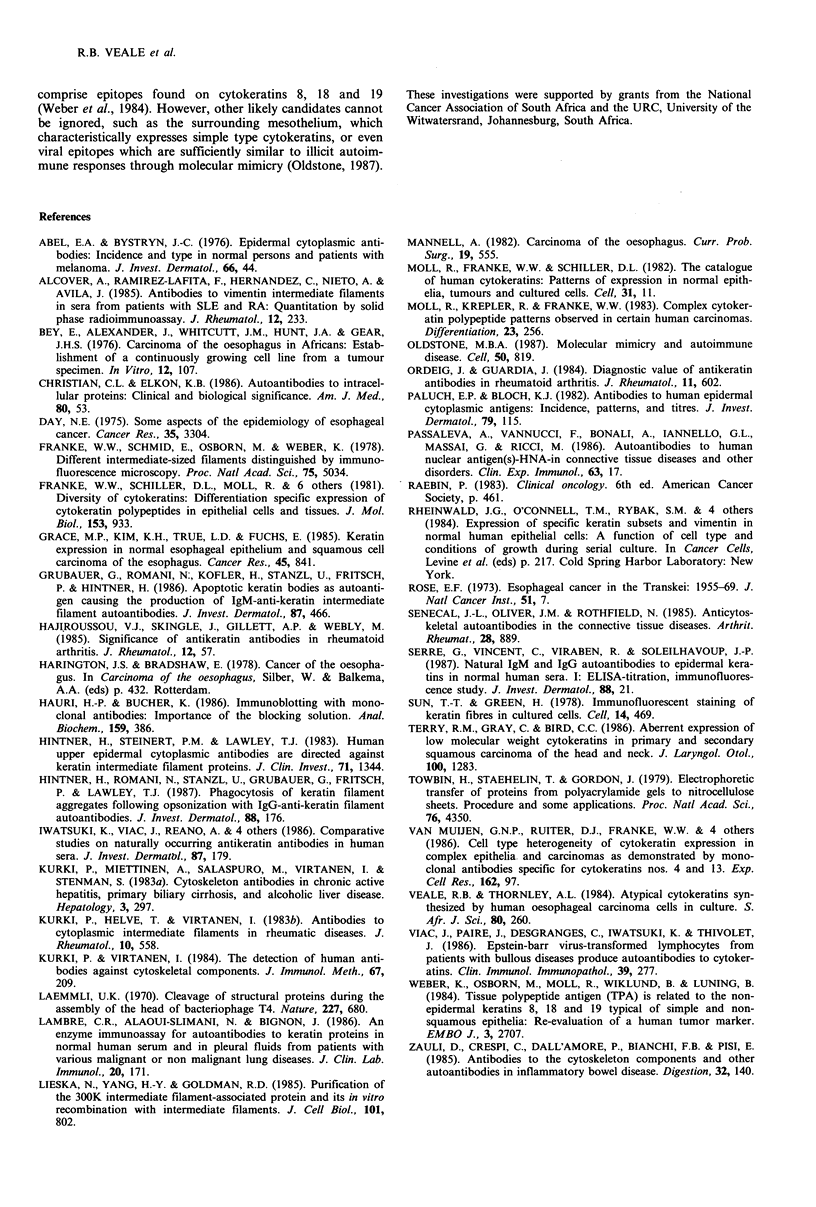# Quantitation of autoantibodies to cytokeratins in sera from patients with squamous cell carcinoma of the oesophagus.

**DOI:** 10.1038/bjc.1988.305

**Published:** 1988-12

**Authors:** R. B. Veale, A. L. Thornley, E. Scott, A. Antoni, I. Segal

**Affiliations:** Department of Zoology, University of the Witwatersrand, South Africa.

## Abstract

**Images:**


Br. J. Cancer (1988), 58, 767–772 © The Macmillan Press Ltd., 1988

### Quantitation of autoantibodies to cytokeratins in sera from patients with squamous cell carcinoma of the oesophagus

R.B. Veale[1], A.L. Thornley[1], E. Scott[1], A. Antoni[2] & I. Segal[2]

[1]*Department of Zoology, University of the Witwatersrand, WITS 2050 and* [2]*Gastroenterology Unit, Baragwanath Hospital, South Africa.*

**Summary** Sera drawn from healthy individuals, patients with squamous cell carcinoma (SCC) of the oesophagus and patients with mild active oesophagitis were examined for autoantibodies to cytoskeletal proteins extracted from the normal oesophageal keratinocyte or from 2 carcinoma cell lines, each of the latter have a simple cytoskeleton.

Using a radioimmunoassay with normal oesophageal cytokeratins as bound antigen, 86 normal, 76 SCC and 14 oesophagitis sera were compared. No significant difference in autoantibody titre was found. When the bound antigen was changed to one containing predominantly simple epithelial cytokeratins a significant increase (32% $P<0.001$) was noted in the SCC category only.

Western blots using simple epithelial cell extracts as antigen revealed autoantibodies to cytokeratins 8, 18 and 19 as well as to one other unidentified protein in most SCC sera, and in some normal sera. Antibodies to cytokeratin 18 predominated.

Normal and SCC sera were applied using indirect immunofluorescent techniques to normal oesophageal keratinocytes, SNO oesophageal SCC cells and HeLa cells grown *in vitro*. Autoantibodies to oesophageal cytokeratins were, with few exceptions, barely detectable. Strong reactions were noted against SNO and HeLa cytoskeletal components, but also against nuclear membrane and nucleolar determinants.

These experiments suggest that raised levels of autoantibodies to certain cytoskeletal and nuclear determinants may be a feature of oesophageal cancer.

An autoimmune response occurs when the immune system is exposed to antigens normally found within the cell. It is a common phenomenon in diseased patients, as well as normal healthy individuals (Christian & Elkon, 1986; Serre *et al.*, 1987; Iwatsuki *et al.*, 1986; Passaleva *et al.*, 1986). Autoantibodies against intracellular antigens can be broadly subdivided into two groups, (a) nuclear and (b) cytoplasmic. The nuclear group comprises autoantibodies to DNA, histones, non-histone proteins and nucleolar antigens. These antibodies could be described as having a loose association with rheumatic and blood diseases (Passaleva *et al.*, 1986; Christian & Elkon, 1986). On the other hand, the cytoplasmic group is primarily represented by autoantibodies against constituents of the three cytoskeletal systems: the microfilaments, microtubules and the intermediate filaments (Alcover *et al.*, 1985; Senecal *et al.*, 1985; Zauli *et al.*, 1985). Circulating antibodies to the microfilaments and microtubules, or actin and tubulin respectively, have been demonstrated in patients with a variety of liver diseases, rheumatoid diseases, SLE and in some other less common syndromes (Kurki *et al.*, 1983*a,b*; Senecal *et al.*, 1985; Zauli *et al.*, 1985).

As the cytokeratins comprise the largest subfamily within the intermediate group of filaments (containing some 20 distinct polypeptides), it is not surprising that autoantibodies to cytokeratins occur in human sera (Iwatsuki *et al.*, 1986; Serre *et al.*, 1987). In fact, autoantibodies to epidermal cytokeratins seem to be a common phenomenon in human sera. Studies have shown that most, though not all, human antiepidermal antibodies are directed against cytokeratin polypeptides characteristic of the basal, suprabasal and stratum corneum cell layers (Abel & Bystryn, 1976; Paluch & Bloch, 1982; Hintner *et al.*, 1983; Iwatsuki *et al.*, 1986).

The synthesis of antibodies to cytokeratins specific to basal or suprabasal epidermal strata appears to be a characteristic of certain epidermal diseases, such as bullous pemphigus (see Viac *et al.*, 1986). However, it has not yet been established whether raised levels of autoantibodies to cytokeratins in human serum are associated with the presence of squamous cell carcinoma (SCC).

At present, little or nothing is known about the specificity of circulating autoantibodies to antigenic components of stratified squamous epithelia other than the epidermis, for example, the cytokeratins of the internal organs such as the oesophagus. It should be noted that the oesophageal epithelium expresses its own distinctive subset of acidic and basic cytokeratin polypeptides (Moll *et al.*, 1982), a pattern which is maintained in the associated neoplasms (Moll *et al.*, 1982; Grace *et al.*, 1985).

Oesophageal SCC has a global distribution but epidemiological studies indicate that, geographically, Africa and in particular South Africa, has the highest incidence (Rose, 1973; Day, 1975; Harington & Bradshaw, 1978).

Lack of a method for early detection has made treatment extremely difficult. Palliation is the only form of management possible in the vast majority of patients (Mannell, 1982). With a view to solving this problem we compared levels of autoantibodies to cytokeratins in sera obtained from patients with oesophageal SCC, or mild active oesophagitis, with sera from individuals clinically free of cancer, using a quantitative radioimmunoassay, immunoblotting and immunofluorescence techniques. We have detected a significant increase in autoantibodies to simple cytokeratins only in those patients with oesophageal SCC.

### Materials and methods

### *Serum samples*

All serum samples were obtained from patients who presented at the Gastroenterology Unit of the Baragwanath Hospital. Informed consent was obtained in each case (Ethics No. 16/2/87). Experiments were performed simultaneously and blind on normal control serum, serum from patients subsequently shown to have mild oesophagitis, and serum from patients with histologically confirmed squamous cell carcinoma (SCC) of the oesophagus. The samples were unmasked at the end of several experimental runs and the results pooled and analysed statistically. Two classes of

Correspondence: R.B. Veale.
Received 1 March 1988; and in revised form, 8 July 1988.

control sera were used in the analysis, designated Control 1 and Control 2. The former group comprised completely healthy volunteers whereas the latter were hospital patients admitted for reasons not in any way related to cancer. Reproducibility between experiments was found to be extremely good both from extract to extract and between earlier and later runs. All of the patients were black and fell within the age range of 39–80 with the mean at 57 years. The male/female ratio in the sample was 5.6:1. The statistical validity of the findings was tested using an unpaired Student's *t* test on a Hewlett Packard 85 bench top computer.

*Extraction of cytokeratin proteins*

Oesophageal cytokeratin polypeptides were obtained by extracting the epithelium scraped from fresh post-mortem oesophagi. Samples rich in simple epithelial type cytokeratin polypeptides were obtained by extracting either the human SNO oesophageal SCC cell line (Bey *et al.*, 1976) or D98 HeLa cells (courtesy of E.J. Stanbridge, University of California, Irvine) both of which were grown *in vitro*. All of the above extracts were produced according to the method of Franke *et al.* (1981). Briefly, confluent cell cultures or epithelial scrapes received a rinse with phosphate buffered saline (PBS) followed by a rinse with TNE buffer (140 mM NaCl, 1 mM EDTA, 10 mM Tris-HCl pH 7.6) containing 1 mM phenyl-methyl-sulphonyl-fluoride (PMSF) and Trasylol (100 kallikrein units/ml). Cultured cells were then scraped off the substrate, collected in TNE buffer and pelleted. The cellular or epithelial pellet was subjected to a two step extraction procedure. Firstly, resuspension in TNE buffer containing 1% Triton X-100 for 5 min, a TNE wash, followed by a second extraction of the pellet in TNE containing 1.5 M KCl and 0.5% Triton X-100 for a period of 20 min. After a further two washes with TNE buffer, the final sediment was taken up in lysis buffer (Laemmli, 1970); and boiled for 5 min. The extracted cytokeratin proteins were analysed by SDS-polyacrylamide gel electrophoresis (SDS-PAGE) in a 12.5% vertical slab gel (Laemmli, 1970).

*Radioimmunoassay*

All radioimmunoassay experiments were performed in triplicate. Nitrocellulose paper discs, 5 mm in diameter (Sartorious SM114), were soaked for 2 h in a 1 mg ml$^{-1}$ solution of cytoskeletal extract (see above). Protein determination performed on the discs after the 2 h soak showed that the discs consistently adsorbed 5 $\mu$g protein. Any remaining background was masked by a 2 h treatment with 'blotto' (5% low-fat dried milk, 0.05% Triton X-100, 0.01% Antifoam A in 50 mM Tris-HCl pH 7.8 (see Hauri & Bucher, 1986) and the discs then incubated in 1:10 dilution of human serum for 1 h. This serum dilution was determined by incubating the discs containing 5 $\mu$g of protein in serially diluted sera in order to identify the saturation level. Serum titre producing maximal binding in 8 randomly chosen sera ranged between dilutions of 1:250 and 1:10. As a result all tests were conducted at the 1:10 dilution. After four washes in blotto (15 min per wash) the discs were incubated in 20 ml of blotto containing 2 $\mu$Ci $^{125}$I-protein A (Amersham) for 1 h. Finally the discs were again washed in blotto as above, rinsed in PBS, air dried and counted in a Packard Autogamma counter.

*Immunoblotting*

Cytokeratin extracts produced as above were separated by 12.5% SDS-PAGE and transferred to nitrocellulose paper at 4°C for 2 h (Towbin *et al.*, 1979), and the background blocked in blotto as above. The nitrocellulose paper was then divided into 1 cm wide strips and each strip incubated in 1:50 serum in blotto over night. Strips were washed in 4 changes of blotto over 2 h before being incubated in $^{125}$I-Protein A for 2 h (1 $\mu$Ci/10 ml in blotto). Finally the strips were again washed in blotto as above, air dried and autoradiographed on Fuji X-ray plate.

*Immunofluorescence*

Immunofluorescence procedures were performed essentially as described by Sun and Green (1978) and Franke *et al.* (1978) using human serum diluted at 1:10. Antibody/antigen complexes were visualised by a two-step method of first reacting with biotinylated goat anti-human IgG (1:250) followed thereafter by binding FITC-conjugated streptavidin (1:200).

### Results

*Radioimmunoassay experiments (RIA)*

Figure 1 shows that the normal oesophageal epithelium expresses 8 cytokeratin polypeptides; four high MW components and four subunits with lower mol. wts (1a track 2, and 1b). Although autoantibodies to oesophageal cytokeratins were present in the serum from normal healthy subjects and patients with oesophageal SCC, there was no significant difference in antibody titre between the groups when the oesophageal cytokeratins were employed as the bound antigen (Figure 2).

A clear, statistically significant ($P<0.001$) difference in autoantibody titre between healthy subjects, and patients with oesophageal SCC was observed in experiments using SNO cell extracts (containing cytokeratins (5), 8, 18 and 19 see Figure 3).

Sera from 14 patients with histologically confirmed mild active oesophagitis were compared with sera from normal subjects, and patients with oesophageal SCC. As can be seen from Figure 3, patients with mild active oesophagitis were no different to healthy subjects with respect to autoantibody titre against SNO extracts.

In order to establish that the increase in autoantibody titre was not perhaps against trace amounts of proteins specific to SNO cells, serum analyses were repeated using extracts from HeLa cells, which express cytokeratin components 8, 18 and vimentin (see Figure 1a track 4 and 1d also Moll *et al.*, 1982). Almost identical values (see Figure 3) were obtained; a significant increase in autoantibody titre was seen to occur in the sera from patients with confirmed oesophageal SCC. This result suggests that the presence of cytokeratins (5), 19 and several distinctive cytoskeletal-associated proteins, which are present in the SNO, but not in the HeLa extracts, played no role in the determination of antibody titre. Likewise, vimentin can be excluded as an autoantigen as it is a major component of the cytoskeleton in HeLa cells but not in the SNO cells. This observation makes clear the distinction, on the basis of autoantibody titre, between SCC and diseases like SLE where the primary autoantigen is vimentin (Alcover *et al.*, 1985).

*Immunofluorescence studies*

The cytokeratin cytoskeletal specificity of the circulating autoantibodies was confirmed for 62 sera by immunofluorescence analyses. Out of the 62 sera only 7 from controls and 11 from patients with oesophageal carcinoma displayed a relatively strong positive staining reaction on normal human oesophageal epithelial cells grown in tissue culture. The fibrous pattern indicative of anticytoskeletal antibodies or a prominent nucleolar staining was observed in these cases (see Figure 4).

Both the number of reactive sera, and the intensity of the staining were increased when the same range of sera were tested on SNO cells. That is, 10 sera from the normal controls produced strongly positive results and similarly, 21 sera from patients with carcinoma of the oesophagus. In most instances the fluorescent patterns produced were of a

**Figure 1** One and two dimensional electrophoretic separations of cytokeratin-enriched preparations from normal oesophageal epithelium and cultured SCC cells. (a) Track 1 – calibration proteins, phosphorylase b (94 kD), bovine serum albumin (67 kD), ovalbumin (43 kD), carbonic anhydrase (30 kD), trypsin inhibitor (21.1 kD); Track 2 – normal adult human oesophageal epithelial scrapes; Track 3 – cultured human oesophageal SNO carcinoma cells; Track 4 – cultured D98 HeLa cells; (b) 2 D normal adult human oesophageal epithelial scrapes; (c) 2 D cultured human oesophageal SNO carcinoma cells; (d) 2 D cultured D98 HeLa cells. Note the absence of component 19 in the HeLa separation but the presence of vimentin (v). The arrow indicates actin. The pH gradient is indicated across the top of the figure (Gel = 12.5%).

filamentous nature although there was also evidence of granular cytoplasmic, nuclear membrane and nucleolar staining (see Figure 4). The characteristic staining patterns of actin and vimentin, which are easily distinguished from the cytokeratin-type, were never observed. A typical actin or vimentin fibrous network was absent when immunofluorescence was performed on D98 HeLa, cells which synthesize comparatively large amounts of vimentin (see Figure 1d).

*Western blotting analyses*

Samples of the same SNO extracts, separated by SDS-PAGE and transferred to nitrocellulose, were used to determine the specificity of the autoantibodies present in a range of sera. Of the 28 sera employed in the blotting experiments 10 examples of the various reactions obtained are shown in Figure 5. All the sera contained autoantibodies which reacted with cytokeratin 18, and seventy percent with both 8 and 18. Sixty percent of the sera tested contained autoantibodies to a third component in addition to the above two, *viz.*, cytokeratin 19. Fairly uncommon was the presence of an antibody to a fourth polypeptide (58 kD mol. wt., possibly cytokeratin 5 which occurs in trace amounts in SNO extracts) which reacted strongly when present. A few of the sera contained antibodies to an additional high mol. wt. component (HMW in Figure 5). This is probably not a cytokeratin since it reacted neither with AE1 nor AE3 monoclonal antibodies to cytokeratin (kindly provided by T.-T. Sun, Department of Dermatology, New York University).

### Discussion

There is only one prior report quantifying serum antikeratin activity in patients with SCC. In this instance Lambre *et al.* (1986) assayed cytokeratin antibodies in sera from patients with SCC of the lung but were unable to detect a significant difference from the mean value for a group of normal controls. Here we show that circulating autoantibodies to the cytokeratins expressed by the stratified squamous oesopha-

**Figure 2** Autoantibody reaction to normal oesophageal epithelial cytokeratins in the sera of normal healthy subjects and patients with oesophageal SCC. Cytokeratins 4, 5, 6, 13, 14/15, 16, (17), (18) and 19 appear in normal oesophageal cytokeratin extracts (see Figure 1a track 2 and 1b). The mean value represents the amount of $^{125}$I protein A-serum autoantibody conjugate bound per $\mu$g of cytokeratin extract. The difference between means ($\triangle$) is uniformly compared with Control 2 (see **Materials and methods**). Ag = extract used as the bound antigen. bkg = the level of background in the radioimmunoassay.

**Figure 3** Autoantibody reaction to cytoskeletal extracts of cultured SCC cells in the sera of normal healthy subjects, patients with oesophageal SCC and patients with mild active oesophagitis. SNO cytoskeletal extracts contain cytokeratins (5), 8, 18 and 19 in almost equal quantities (see Figure 1a track 3 and 1c). HeLa extracts contain cytokeratins 8 and 18 together with large quantities of vimentin (v) intermediate filament protein (see Figure 1a track 4 and 1d). The mean value represents the amount of $^{125}$I protein A-serum autoantibody conjugate bound per $\mu$g of cytoskeletal extract. The difference between means ($\triangle$) is uniformly compared with Control 2 (see **Materials and methods**). Ag = extract used as the bound antigen. bkg = the level of background in the radioimmunoassay.

**Figure 4** Indirect immunofluorescent staining of cultured cells. a, b, c: Cultured normal human oesophageal epithelial cells. Note the different staining patterns obtained with different sera; a – the most common staining pattern being distinctly fibrous with discernible desmosomal structures; b – nucleolar staining; c – nuclear staining. d, e, f: Cultured human oesophageal SNO carcinoma cells. Again different staining patterns were obtained with the most common being the fibrous meshwork typical of cytoskeletal staining. The distinctive staining patterns of vimentin and/or actin were absent. Bar = 20 $\mu$m.

**Figure 5** One dimensional electrophoretic separations of cytokeratin-enriched preparations from SNO oesophageal carcinoma cells using serum from patients with oesophageal SCC. Track 1 – nitrocellulose paper showing the electrophoretically transferred cytoskeletal polypeptides stained with fast green; Tracks 2–11 – nitrocellulose-bound SNO carcinoma cell cytoskeletal separation reacted with various sera from patients with oesophageal SCC, conjugated with $^{125}I$ protein-A and visualized by autoradiography. Note that autoantibodies to cytokeratin polypeptide 18 occur most commonly in the sera from these patients followed by antibodies against polypeptides 8 and 19. Note also the relatively common appearance of a high mol. wt. (HMW) component as yet unidentified.

geal epithelium are present in the serum of normal subjects and patients with SCC of the oesophagus. Using a solid phase radioimmunoassay, and the set of normal oesophageal cytokeratins (polypeptides nos. 4, 5, 6, 13, 14/15, 16 and 19) as the bound antigen, we found no significant difference in autoantibody titre (Figure 2). An interesting and unexpected finding was that the autoantibody titre was 32% higher in patients with oesophageal SCC, when SNO or HeLa extracts were employed as the antigen. These extracts contain cytokeratins 8, 18, and 19 or 8, and 18 respectively. Apparently trace amounts of cytokeratin 5 (SNO cells) and large concentrations of vimentin (HeLa cells) played an insignificant role in the increased antibody binding (see Figure 3). This study supports the idea that serum autoantibodies to some types of intermediate filaments can be quantified and therefore are useful indicators of disease. Alcover *et al.* (1985) have shown some time ago that SLE is linked to a raised autoantivimentin titre.

Several authors have postulated that mild active oesophagitis precedes the onset of oesophageal SCC (Mannell, 1982). The data presented in Figure 3 show that there is no difference in the titre of autoantibodies to components of the simple cytoskeleton when normal, healthy controls, and patients with histologically confirmed mild active oesophagitis were compared. These observations lead one to conclude that either mild active oesophagitis may be a condition unconnected with oesophageal SCC, or that there is an intermediate step which hitherto has escaped detection. It is important to note that patients with moderate oesophagitis, which one has been led to suspect as the next step in the progression towards SCC (see Mannell, 1982) are normally very rarely detected. We were able to test only one such case in this study (1/14 = 7.1%) the result of which was indistinguishable from the mild cases. Similarly, adenocarcinoma of the oesophagus forms less than five percent of the cases presenting at Baragwanath Hospital. Our data are as yet insufficient to say whether adenocarcinoma and SCC can be distinguished on the basis of this radioimmunoassay. To date there is no reliable technique that distinguishes between mild active oesophagitis and the preneoplastic state, or stages thereof, as in cancer of the cervix (Raebin, 1983). Clearly every avenue should be thoroughly explored which could possibly facilitate a more accurate early diagnosis of the disease.

Western blotting experiments performed using the sera from the patients with oesophageal SCC showed that, although antibodies to each of the simple epithelial cytokeratins were present in one or another serum, antibodies to polypeptide 18 were present throughout (see Figure 5). This is the first report showing an increased autoantibody titre to a particular cytokeratin in patients with SCC. Future studies will show whether autoantibodies to cytokeratin 18 are associated only with oesophageal SCC or with a variety of unrelated disorders, and perhaps provide an explanation as to why cytokeratin 18 is more antigenic than other similar proteins.

In addition to the identifiable cytokeratin polypeptides, there was at least one other immunoreactive non-cytokeratin protein (A. Thornley, unpublished results) present in the high-salt cytoskeletal residue (see HMW in Figure 5). The identity of these proteins is at present unknown but it has been shown that cytoskeletal-associated proteins frequently co-purify with intermediate filaments (Lieska *et al.*, 1985).

Autoantibodies to cytoskeletal components such as actin, vimentin, desmin and cytokeratin have been described in diseases such as SLE, rheumatoid arthritis, liver diseases and bullous diseases (Kurki *et al.*, 1983*a*,*b*; Ordeig & Guardia, 1984; Senecal *et al.*, 1985; Alcover *et al.*, 1985; Hajiroussou *et al.*, 1985). Patients with oesophageal SCC may have raised levels of autoantibodies to nuclear as well as cytoskeletal structures. Nuclear, nuclear membrane and in particular nucleolar staining were striking features of many of the sera tested on cultures of normal oesophageal epithelial cells or SNO cells (Figure 4).

Several authors subscribe to the idea that anticytokeratin antibodies are formed in response to epithelial cell injury or death (Kurki & Virtanen, 1984; Zauli *et al.*, 1985; Grubauer *et al.*, 1986; Hintner *et al.*, 1987). A similar event could account for our results. The most difficult observation to explain is why antibodies should form predominantly to the simple epithelial cytokeratins, and to number 18 in particular. Previously we reported atypical cytokeratin expression *in vitro* by a human oesophageal SCC cell line (Veale & Thornley, 1984). This involved the synthesis of a cytokeratin profile which was distinctly simple epithelial-like in nature consisting of polypeptides 8, 18 and 19 with trace amounts of 5 also present (see Figure 1a track 3 and 1c, confirmed using antibodies AE1 and AE3, Thornley unpublished results). Terry *et al.* (1986) have now reported the presence of simple cytokeratins in primary oesophageal tumours, but this is not true for SCCs in general (Moll *et al.*, 1983; Rheinwald *et al.*, 1984) or oesophageal SCCs in particular (Grace *et al.*, 1985; Van Muijen *et al.*, 1986). Nevertheless, it is feasible that the oesophageal SCC tumour itself is the source of immunoreactive intracellular antigens, possibly in the form of tissue polypeptide antigen (TPA), now known to comprise epitopes found on cytokeratins 8, 18 and 19 (Weber et al., 1984). However, other likely candidates cannot be ignored, such as the surrounding mesothelium, which characteristically expresses simple type cytokeratins, or even viral epitopes which are sufficiently similar to illicit autoimmune responses through molecular mimicry (Oldstone, 1987).

These investigations were supported by grants from the National Cancer Association of South Africa and the URC, University of the Witwatersrand, Johannesburg, South Africa.

### References

ABEL, E.A. & BYSTRYN, J.-C. (1976). Epidermal cytoplasmic antibodies: Incidence and type in normal persons and patients with melanoma. *J. Invest. Dermatol.*, **66**, 44.

ALCOVER, A., RAMIREZ-LAFITA, F., HERNANDEZ, C., NIETO, A. & AVILA, J. (1985). Antibodies to vimentin intermediate filaments in sera from patients with SLE and RA: Quantitation by solid phase radioimmunoassay. *J. Rheumatol.*, **12**, 233.

BEY, E., ALEXANDER, J., WHITCUTT, J.M., HUNT, J.A. & GEAR, J.H.S. (1976). Carcinoma of the oesophagus in Africans: Establishment of a continuously growing cell line from a tumour specimen. *In Vitro*, **12**, 107.

CHRISTIAN, C.L. & ELKON, K.B. (1986). Autoantibodies to intracellular proteins: Clinical and biological significance. *Am. J. Med.*, **80**, 53.

DAY, N.E. (1975). Some aspects of the epidemiology of esophageal cancer. *Cancer Res.*, **35**, 3304.

FRANKE, W.W., SCHMID, E., OSBORN, M. & WEBER, K. (1978). Different intermediate-sized filaments distinguished by immunofluorescence microscopy. *Proc. Natl Acad. Sci.*, **75**, 5034.

FRANKE, W.W., SCHILLER, D.L., MOLL, R. & 6 others (1981). Diversity of cytokeratins: Differentiation specific expression of cytokeratin polypeptides in epithelial cells and tissues. *J. Mol. Biol.*, **153**, 933.

GRACE, M.P., KIM, K.H., TRUE, L.D. & FUCHS, E. (1985). Keratin expression in normal esophageal epithelium and squamous cell carcinoma of the esophagus. *Cancer Res.*, **45**, 841.

GRUBAUER, G., ROMANI, N., KOFLER, H., STANZL, U., FRITSCH, P. & HINTNER, H. (1986). Apoptotic keratin bodies as autoantigen causing the production of IgM-anti-keratin intermediate filament autoantibodies. *J. Invest. Dermatol.*, **87**, 466.

HAJIROUSSOU, V.J., SKINGLE, J., GILLETT, A.P. & WEBLY, M. (1985). Significance of antikeratin antibodies in rheumatoid arthritis. *J. Rheumatol.*, **12**, 57.

HARINGTON, J.S. & BRADSHAW, E. (1978). Cancer of the oesophagus. In *Carcinoma of the oesophagus*, Silber, W. & Balkema, A.A. (eds) p. 432. Rotterdam.

HAURI, H.-P. & BUCHER, K. (1986). Immunoblotting with monoclonal antibodies: Importance of the blocking solution. *Anal. Biochem.*, **159**, 386.

HINTNER, H., STEINERT, P.M. & LAWLEY, T.J. (1983). Human upper epidermal cytoplasmic antibodies are directed against keratin intermediate filament proteins. *J. Clin. Invest.*, **71**, 1344.

HINTNER, H., ROMANI, N., STANZL, U., GRUBAUER, G., FRITSCH, P. & LAWLEY, T.J. (1987). Phagocytosis of keratin filament aggregates following opsonization with IgG-anti-keratin filament autoantibodies. *J. Invest. Dermatol.*, **88**, 176.

IWATSUKI, K., VIAC, J., REANO, A. & 4 others (1986). Comparative studies on naturally occurring antikeratin antibodies in human sera. *J. Invest. Dermatol.*, **87**, 179.

KURKI, P., MIETTINEN, A., SALASPURO, M., VIRTANEN, I. & STENMAN, S. (1983*a*). Cytoskeleton antibodies in chronic active hepatitis, primary biliary cirrhosis, and alcoholic liver disease. *Hepatology*, **3**, 297.

KURKI, P., HELVE, T. & VIRTANEN, I. (1983*b*). Antibodies to cytoplasmic intermediate filaments in rheumatic diseases. *J. Rheumatol.*, **10**, 558.

KURKI, P. & VIRTANEN, I. (1984). The detection of human antibodies against cytoskeletal components. *J. Immunol. Meth.*, **67**, 209.

LAEMMLI, U.K. (1970). Cleavage of structural proteins during the assembly of the head of bacteriophage T4. *Nature*, **227**, 680.

LAMBRE, C.R., ALAOUI-SLIMANI, N. & BIGNON, J. (1986). An enzyme immunoassay for autoantibodies to keratin proteins in normal human serum and in pleural fluids from patients with various malignant or non malignant lung diseases. *J. Clin. Lab. Immunol.*, **20**, 171.

LIESKA, N., YANG, H.-Y. & GOLDMAN, R.D. (1985). Purification of the 300K intermediate filament-associated protein and its *in vitro* recombination with intermediate filaments. *J. Cell Biol.*, **101**, 802.

MANNELL, A. (1982). Carcinoma of the oesophagus. *Curr. Prob. Surg.*, **19**, 555.

MOLL, R., FRANKE, W.W. & SCHILLER, D.L. (1982). The catalogue of human cytokeratins: Patterns of expression in normal epithelia, tumours and cultured cells. *Cell*, **31**, 11.

MOLL, R., KREPLER, R. & FRANKE, W.W. (1983). Complex cytokeratin polypeptide patterns observed in certain human carcinomas. *Differentiation*, **23**, 256.

OLDSTONE, M.B.A. (1987). Molecular mimicry and autoimmune disease. *Cell*, **50**, 819.

ORDEIG, J. & GUARDIA, J. (1984). Diagnostic value of antikeratin antibodies in rheumatoid arthritis. *J. Rheumatol.*, **11**, 602.

PALUCH, E.P. & BLOCH, K.J. (1982). Antibodies to human epidermal cytoplasmic antigens: Incidence, patterns, and titres. *J. Invest. Dermatol.*, **79**, 115.

PASSALEVA, A., VANNUCCI, F., BONALI, A., IANNELLO, G.L., MASSAI, G. & RICCI, M. (1986). Autoantibodies to human nuclear antigen(s)-HNA-in connective tissue diseases and other disorders. *Clin. Exp. Immunol.*, **63**, 17.

RAEBIN, P. (1983). *Clinical oncology*. 6th ed. American Cancer Society, p. 461.

RHEINWALD, J.G., O'CONNELL, T.M., RYBAK, S.M. & 4 others (1984). Expression of specific keratin subsets and vimentin in normal human epithelial cells: A function of cell type and conditions of growth during serial culture. In *Cancer Cells*, Levine *et al.* (eds) p. 217. Cold Spring Harbor Laboratory: New York.

ROSE, E.F. (1973). Esophageal cancer in the Transkei: 1955–69. *J. Natl Cancer Inst.*, **51**, 7.

SENECAL, J.-L., OLIVER, J.M. & ROTHFIELD, N. (1985). Anticytoskeletal autoantibodies in the connective tissue diseases. *Arthrit. Rheumat.*, **28**, 889.

SERRE, G., VINCENT, C., VIRABEN, R. & SOLEILHAVOUP, J.-P. (1987). Natural IgM and IgG autoantibodies to epidermal keratins in normal human sera. I: ELISA-titration, immunofluorescence study. *J. Invest. Dermatol.*, **88**, 21.

SUN, T.-T. & GREEN, H. (1978). Immunofluorescent staining of keratin fibres in cultured cells. *Cell*, **14**, 469.

TERRY, R.M., GRAY, C. & BIRD, C.C. (1986). Aberrent expression of low molecular weight cytokeratins in primary and secondary squamous carcinoma of the head and neck. *J. Laryngol. Otol.*, **100**, 1283.

TOWBIN, H., STAEHELIN, T. & GORDON, J. (1979). Electrophoretic transfer of proteins from polyacrylamide gels to nitrocellulose sheets. Procedure and some applications. *Proc. Natl Acad. Sci.*, **76**, 4350.

VAN MUIJEN, G.N.P., RUITER, D.J., FRANKE, W.W. & 4 others (1986). Cell type heterogeneity of cytokeratin expression in complex epithelia and carcinomas as demonstrated by monoclonal antibodies specific for cytokeratins nos. 4 and 13. *Exp. Cell Res.*, **162**, 97.

VEALE, R.B. & THORNLEY, A.L. (1984). Atypical cytokeratins synthesized by human oesophageal carcinoma cells in culture. *S. Afr. J. Sci.*, **80**, 260.

VIAC, J., PAIRE, J., DESGRANGES, C., IWATSUKI, K. & THIVOLET, J. (1986). Epstein-barr virus-transformed lymphocytes from patients with bullous diseases produce autoantibodies to cytokeratins. *Clin. Immunol. Immunopathol.*, **39**, 277.

WEBER, K., OSBORN, M., MOLL, R., WIKLUND, B. & LUNING, B. (1984). Tissue polypeptide antigen (TPA) is related to the non-epidermal keratins 8, 18 and 19 typical of simple and non-squamous epithelia: Re-evaluation of a human tumor marker. *EMBO J.*, **3**, 2707.

ZAULI, D., CRESPI, C., DALL'AMORE, P., BIANCHI, F.B. & PISI, E. (1985). Antibodies to the cytoskeleton components and other autoantibodies in inflammatory bowel disease. *Digestion*, **32**, 140.